# Dietary and Lifestyle Strategies for Obesity

**DOI:** 10.3390/nu16162714

**Published:** 2024-08-15

**Authors:** Thomas M. Barber, Stefan Kabisch, Andreas F. H. Pfeiffer, Martin O. Weickert

**Affiliations:** 1 Warwickshire Institute for the Study of Diabetes, Endocrinology and Metabolism, University Hospitals Coventry and Warwickshire, Clifford Bridge Road, Coventry CV2 2DX, UK; t.barber@warwick.ac.uk; 2 Division of Biomedical Sciences, Warwick Medical School, University of Warwick, Coventry CV1 5FB, UK; 3 NIHR CRF Human Metabolism Research Unit, University Hospitals Coventry and Warwickshire, Clifford Bridge Road, Coventry CV2 2DX, UK; 4 Deutsches Zentrum für Diabetesforschung e.V., Geschäftsstelle am Helmholtz-Zentrum München, Ingolstädter Landstraße, 85764 Neuherberg, Germany; stefan.kabisch@charite.de (S.K.); afhp@charite.de (A.F.H.P.); 5 Department of Endocrinology, Diabetes and Nutrition, Campus Benjamin Franklin, Charité University Medicine, Hindenburgdamm 30, 12203 Berlin, Germany; 6 Centre for Sport, Exercise and Life Sciences, Faculty of Health & Life Sciences, Coventry University, Coventry CV1 5FB, UK

**Keywords:** obesity, diet, sleep, appetite, fibre, carbohydrate, ultra-processed foods

## Abstract

The prevalence of obesity globally has tripled over the last half century, and currently affects around 650 million adults and 340 million children and adolescents (ages 5–19 years). Obesity contributes towards >50 co-morbidities and premature mortality. Obesity is a highly stigmatised condition that is associated with much mental and emotional distress and dysfunction. Thus, obesity is a major contributor to healthcare expenditure globally. Traditionally, the management of obesity stratifies into three major groups that include metabolic (bariatric) surgery, pharmacotherapies, and lifestyle (primarily dietary) strategies. Although listed as a separate category, dietary strategies for obesity remain a central component of any management plan, and often complement other surgical and pharmacotherapeutic options. Indeed, the effectiveness of any management approach for obesity relies upon successful behavioural changes, particularly relating to eating behaviours. In this concise review, we explore the foundational pillars of dietary strategies for obesity: sleep, listening, routine, de-stressing and optimisation of social conditions. We then discuss the importance of balancing dietary macronutrients (including dietary fibre, carbohydrates, protein and ultra-processed foods [UPFs]) as a key dietary strategy for obesity. Although we focus on general principles, we should provide bespoke dietary strategies for our patients, tailored to their individual needs. Rather than judging the utility of a diet based simply on its associated magnitude of weight loss, we should adopt a more holistic perspective in which a dietary strategy is valued for its overall health benefits, including the nurturing of our gut microbiota, to enable them to nurture and protect us.

## 1. Introduction

The global prevalence of obesity has tripled over the last half century, and currently affects around 650 million adults and 340 million children and adolescents (ages 5–19 years) [1,2]. The importance of obesity as a clinical entity and disease stems from its close association with >50 co-morbidities, most notably type 2 diabetes mellitus (T2D) and other conditions that typify metabolic dysfunction (hypertension, dyslipidaemia, metabolic-associated fatty liver disease [MAFLD], obstructive sleep apnoea [OSA]), biomechanical disorders, respiratory, cardiovascular, and psychiatric conditions, and some malignancies [3]. Obesity (especially severe obesity with BMI > 40 Kgm^−2^) is also associated with premature mortality [4,5]. Obesity is also a highly stigmatised condition [6], and the negative impact of this, combined with multiple co-morbidities and impairment of wellbeing [7], results in a diminishment of psycho-social functioning [8] and work productivity [9]. Inevitably, these factors combine to promote obesity (both directly and indirectly) as a major contributor to global healthcare expenditure [10].

It is beyond the scope of this concise review to provide a detailed overview of the pathogenesis of obesity, which has been outlined recently [11,12]. Although dietary and lifestyle factors indubitably contribute towards weight gain and the development of obesity, these behaviours need to be considered in the context of complex interlinks with underlying genetic factors. Recent genome-wide association studies (GWAS) have transformed our understanding of the pathogenesis of obesity, and data from nearly 340,000 subjects reveal that 97 loci are associated with BMI [11]. These loci affect genes that are expressed at the level of the central nervous system. Furthermore, monogenic forms of obesity implicate proteins expressed within the hypothalamic appetite regulating pathway (with the notable exception of leptin) [11]. These insights promote obesity as a neurobehavioral disorder [11]. To add to this complexity, epigenetic factors (influencing gene expression rather than the structure of the gene itself), are also implicated in obesity development, and should form a key focus of future obesity-related research.

Broadly, our current management strategies for obesity span three major groups: lifestyle change [13]; pharmacotherapies [14,15], and bariatric (metabolic) surgery [16,17]. Traditionally, these management strategies are usually applied sequentially, with bariatric surgical approaches reserved as a last resort. However, with the rise of the incretin-based pharmacotherapies, including dual and triple incretin agonists and their associated remarkable weight-loss effects (almost now equivalent to some forms of bariatric surgery) [14,15], it seems likely that future treatment algorithms for obesity will place less emphasis on the relative positioning of each management option generally, but rather focus on the needs of individuals. Furthermore, with the dawn of a new era of cardiovascular outcome trials for obesity-focused therapies, including the SELECT trial for semaglutide [18], it seems logical that future management strategies for obesity management should place more emphasis on the potential for suggested therapeutic strategies to improve overall cardiometabolic health rather than solely to provide weight-loss effects. Such a sea-change in mindset from BMI to cardiometabolic centricity when considering therapeutic strategies for obesity should also catalyse an important and timely transition in our approach to the diagnosis of obesity, with reduced reliance on BMI as an anachronistic and inherently flawed criterion, and more emphasis on the cardiometabolic sequelae of excessive adiposity. 

Dietary modification in obesity usually falls under the umbrella of ‘lifestyle change’. Despite such a categorisation, dietary modification forms an essential component of any strategy in the management of obesity. This includes, for example, compliance with dietary rules and changes prior to and following bariatric surgery, and the need to adjust caloric intake according to diminished appetite with the administration of incretin-based pharmacotherapies. Importantly, not all dietary modification is restrictive, such as the importance of adequate intake of vegetables. As such, dietary strategies are integral to obesity management and deserve special focus. Furthermore, dietary strategies in obesity depend on behavioural change at an individual level [19]. Unfortunately, the popular media, influencers and self-proclaimed nutrition experts are awash with recommendations for fad diets as a ‘quick fix’ solution for obesity, with little if any supporting scientific evidence [20,21,22]. 

Understandably, people living with obesity (the majority of whom are not fortunate enough to have received focused and individualised dietary advice from a specialist weight-management dietician) are often confused regarding which diet to follow. Furthermore, failure to achieve weight loss with a specific diet may enhance negative thoughts and attitudes towards obesity generally and have a de-motivating effect on any future attempt at losing weight. This confusion regarding which dietary strategy to follow was compounded for many years by an emphasis on ‘low-fat’ diets [23] from governments and healthcare professionals alike, with a compensatory need for increased sugar content of processed foods (to facilitate palatability) as an important contributor to the current global obesity problem. Fortunately, in recent times, there has been a shift in focus from ‘low fat’ to ‘low-carbohydrate’ foods [24], with associated ‘sugar-tax’ and limitations on food advertising for children, which are excellent initiatives that can only help to improve the health of our society, and particularly our children, which should be prioritized. 

In this concise narrative review, we consider the key principles of dietary strategies for the management and prevention of obesity. There is a vast literature on this topic. Whilst it is beyond the scope of our review to consider all aspects of lifestyle, it is apt to acknowledge the importance of avoidance of sedentariness and the clear health benefits of regular physical activity. The important role of regular physical activity in the prevention and management of obesity has been expertly reviewed recently elsewhere [25]. Accordingly, here we focus on key insights relating to dietary strategies for obesity (summarized in Table 1), rather than providing an exhaustive exposition of the entire topic. Broadly, we explore the subject from three separate angles: (i) building the foundations for dietary modification; (ii) balancing macronutrients; (iii) dietary restrictions. We conclude our review with a suggested strategy for dietary modification to facilitate weight loss, based on the scientific data. Although our review focuses primarily on the management of obesity through the promotion of a healthy lifestyle and diet, the strategies outlined could equally apply to the effective prevention of weight gain and subsequent obesity. As such, the principles outlined here should resonate with a population-wide approach for the effective management and prevention of obesity. 

## 2. Methodology

We used PubMed as a search tool. We used the terms ‘obesity’, ‘diet’, ‘sleep’, ‘appetite’, ‘fibre’, ‘carbohydrate’, and ‘ultra-processed foods’ to provide relevant articles for our narrative review. We only used papers written in English. We prioritized papers that have been published more recently without restricting published papers according to date of publication. Due to the concise nature of our review, we did not include an exhaustive list of references.

## 3. Building the Foundations for Dietary Modification

The most important part of any house-build is the first step: the foundations. Likewise, the successful implementation of dietary modification necessitates the initial establishment and maintenance of a firm foundation. Attempting dietary modification without due attention to foundations can prove futile and have a de-motivating effect. It is difficult to overstate the importance of foundations in the context of dietary modification. Unfortunately, this crucial step is often overlooked, which can contribute towards failed attempts at dietary modification. In this section, we focus on five pillars of lifestyle that represent key structures and form foundations. Here, we consider each of these pillars as sub-sections and emphasize that these should each be optimised prior to any attempt at dietary modification. 

### 3.1. Sleeping 

There is an Irish proverb that says ‘*a good laugh and a long sleep are the best cures in the doctor’s book*’ [26]. Sleep deprivation negatively affects virtually every aspect of physiology and is linked to much morbidity and even premature mortality [26]. There is a clear and well-established link between sleep and appetite. This topic has been comprehensively reviewed recently, with insights derived from epidemiological and animal- and human-based studies [27]. Sleep deprivation is associated with changes in appetite-regulating hormones such as ghrelin, leptin, and insulin [27]. An intervention study on healthy men with just 2 days of sleep curtailment revealed significant changes in serum ghrelin and leptin, and enhanced appetite especially for highly calorific foods with high carbohydrate content [28]. 

Over the last 100 years, there has been a reduction in our sleep duration. Incredibly, in the early 20th century, adults slept on average around 9 h per day, and this reduced to around 7 h of sleep per day in the 1980s [26,29]. In 2010, 30% of adults slept <7 h each day [26,29]. The prevalence of sleep deprivation that causes excessive daytime sleepiness is between 9 and 24% [26]. There are myriad factors that have contributed towards sleep deprivation in the modern era, including increasing demands on our time, a 24-h society with instant access to all manner of services, increased use of electronic devices, shift work and increased stress [26]. Of note, our availability as individuals has transformed radically in recent times with the widespread use of electronic devices, which seem to be ubiquitous and omnipresent and follow us everywhere we go, often including to bed. Despite the benefits, one downside of our modern-day, highly connected and ‘24-h instant access’ society is the inherent intrusiveness into our daily lives, which can include intrusion into our cherished and essential sleeping time, with adverse effects on both the duration and quality of sleep. 

Unsurprisingly, sleep deprivation stymies any attempt at dietary modification. In one randomized cross-over study on overweight adults who underwent 14 days of moderate caloric restriction with 8.5 or 5.5 h of nighttime sleep opportunity, sleep curtailment reduced the proportion of weight lost as fat by 55% and increased the loss of fat-free body mass by 60% [30]. Sleep curtailment also increased the neuroendocrine adaptation to caloric restriction, with enhanced hunger and a relative reduction in fat oxidation [30].

The National Institutes of Health recommends that as adults, we should get 7–8 h of sleep each day [26,31]. Many of us are sleep-deprived and get appreciably less sleep than the recommended sleep duration. Indeed, sleep deprivation is a likely contributor to the global obesity problem. It is beyond the scope of this review to discuss the guidelines for sleep optimisation which have been reviewed elsewhere [32]. As a first step in establishing the foundations for dietary modification, we should ensure the implementation of such guidance and discuss strategies by which our patients can achieve and maintain sleep sufficiency and quality. This is likely to include a discussion of daily routine, the optimisation of a regular sleep–wake cycle, and the referral to sleep centres to diagnose and manage any sleep-related disorder such as insomnia and/or obstructive sleep apnoea that may negatively impact sleep duration and quality. Indeed, many patients with sleep insufficiency report long-lasting insomnia [33]. In addition to the impact of sleep deprivation on enhanced appetite and unfavourable metabolic processes, nutrition also influences sleeping wellness and hormonal and inflammatory pathways that can in turn influence both sleep duration and quality [34]. Therefore, the maintenance of sleep sufficiency and quality is facilitated by healthy dietary modification and vice versa.

### 3.2. Listening

In our modern-day world, it seems that everyone should have an opinion. The rise of social media encourages us all to share our opinions with the rest of the world, and positively reinforces such behaviour through ‘likes’. With such an emphasis on voice, it seems that the art of active listening has been forgotten. This seems ironic given that active listening forms a foundation for good communication. In the same way, we are so distracted by the outside world and with voicing our own opinions that the art of active listening seems to have been forgotten. Externally, we should all learn not to listen to everyone who claims to be an ‘expert’. We need to be sure who really is an expert, and then listen actively to their expertise. Internally, we need to re-learn how to listen and respond to the interoceptive signals that reach our brain, that inform us continuously to our current state of wellbeing, and alert us to any emergent problem (such as localised pain, or increased appetite for food). Like any skill, the art of active listening to one’s own mind and body is one that needs to be learnt and nurtured. In our own hospital-based obesity service, we innovated the art of mindfulness (a form of active internal listening) into our own tier-3 pathway, and demonstrated positive effects on eating behaviours and also improvements in self-esteem and self-confidence [35].

To facilitate active listening, we need to de-clutter our lives and provide ourselves with time and space. With so many demands on us, effective time management can seem like a challenge in which the aim is to cram as much into our lives as possible. However, such an approach can have a negative impact on our ability to listen. Importantly, we need to enable ourselves to listen to our internal requirements for food through recognising appetitive signals. Unfortunately, eating behaviours are often habituated, with many of us regimented to three meals per day. At other times, we may eat to experience the hedonic effects of food in the context of boredom or dysthymia, with such behaviour often being ‘mindless’ [36]. Furthermore, many social events are associated with food, and social cues to group eating (particularly from one’s inner social group) can be very strong and difficult to resist [37]. Eating behaviours can also be influenced by social media [38]. Whilst it may be impossible (and undesirable) for us only ever to eat in response to hunger cues, there is a general need within our modern-day society for us to listen more to our internal appetitive signals and try to eat in response to hunger (analogous to drinking fluids to quench thirst) rather than predominantly for other reasons. If this change in mindset means that we occasionally skip a meal because we are not hungry, then we can take solace from the fact that we did not evolve to eat three meals every day, and our physiology is much better suited to some periods of relative caloric omission [39]. 

One limitation and caution of listening to one’s own appetitive signals is that this can be seen by some as an open invitation for ‘intuitive eating’. Although mainly observational studies have shown benefits from ‘intuitive eating’, it should be recognised that those who eat intuitively tend to be healthy and adopt heathy lifestyles generally [40]. Therefore, such data may simply reflect reverse causality. Unfortunately, intervention studies that specifically assess the effects of intuitive eating are rare, with most data on this topic stemming from RCTs, in which ‘intuitive eating’ is often applied to a control group without intervention [40]. This in turn can result in biased data that skew outcomes from a plethora of dietary interventions. Unfortunately, several pseudo-scientific conspiracy theorists claim that ‘intuitive eating’ is THE natural and pure way to eat, and that virtually every scientific recommendation is just a hoax to manipulate our minds and force veganism or other dietary changes into our societies. Hypocritically, such conspiracy theorists typically criticize scientific guidelines based on evidence from cohort studies, whilst themselves advocating ‘intuitive eating’ based on the same types of studies. A further caveat of active listening to one’s own internal state is that we often lack interoceptive signals of metabolic dysfunction: put simply, we can feel well and yet still harbour import obesity-related conditions, which are generally under-diagnosed. Furthermore, a sub-diagnostic labelling of ‘metabolically healthy obesity’ (MHO) can lull us into a false sense of security and contribute towards a delay in screening for obesity-related conditions (including for example, obesity-related malignancies), therefore hampering the effective prevention and management of such conditions.

### 3.3. Routine

Many of us have busy and sometimes chaotic lives. The challenges of holding a job, rearing children, sustaining friendships, caring for loved ones, managing mental health problems, and just trying to be the best version of ourselves can be demanding and exhausting. When considering foundational optimization, we need to be realistic about what can be achieved. Setting goals that are unrealistic and/or unachievable will likely have a negative and de-motivating effect. Conversely, setting realistic goals can inspire and motivate. The achievement of goals generally requires a degree of order and routine to our lives. In this context, we use the term ‘routine’ mainly to illustrate a regular sleep–wake cycle rather than to necessarily refer to a regimented eating pattern that is based on timing rather than appetitive signals, as discussed above. Unfortunately, the establishment and maintenance of routine has always been a challenge for humans, given the unpredictability of life. Centuries ago, wars, famines, pandemics, and ‘regular crimes’ struck people and interrupted the routine of life far more frequently than in our relatively pacified and modern-day world. Personal crises in the past often resulted in immediate death, or severely compromised life and routine with disease and/or starvation. In our modern-day world, the routine of life is still compromised but by a different set of challenges. Personal crises nowadays can result in weight gain, obesity, and metabolic dysfunction, resulting from the ubiquitous and continuous availability of cheap, storable and rewarding food, concurrent with limited availability, affordability, and societal acceptability of healthy foods.

The unpredictability of modern-day life, and the intrusiveness of our technologies on us as individuals can make routine seem impossible at times. Furthermore, whilst many of us have a regular quotidian routine, this can be severely compromised by a plethora of modern-day conditions that stymie such an ideal, such as attention deficit hyperactivity disorder, depression, anxiety, schizophrenia, myalgic encephalomyelitis, chronic fatigue syndrome and long COVID [41]. Such conditions are often associated with sleep and circadian rhythm disturbance, that in turn is linked with insulin resistance [42] and other key components of the metabolic syndrome [43,44]. Whilst some disturbance of daily routine is perhaps an inevitable consequence of modern daily living, we should at least strive to establish and maintain the routine of a regular sleep–wake cycle where possible, with constant bed and wake-up times. Furthermore, we should adjust our daily activities to facilitate such routines with, for example, avoidance of physical exercise in the run-up to bedtime. Interestingly, although it is generally accepted that we should avoid eating in the hour prior to sleep, a recent study on the effects of timing of evening dinner in relation to sleep suggests that eating 1 h prior to sleep does not result in significant adverse changes in overnight sleep architecture [45]. We should adopt an individualised approach to lifestyle recommendations, but one that is also based on the available evidence. 

### 3.4. De-Stressing

We use the term ‘stress’ here to refer to a state of mental or emotional strain or tension resulting from adverse or demanding circumstances. Stress is an umbrella term and incorporates a whole range of scenarios. It seems that stress is integral to our lives. Given the complexity of our world, and the dynamic nature of life and its vagrant trajectories, it seems almost inevitable that all of us will experience stress at some stage, and many of us recurrently and chronically. In the context of dietary strategies and obesity, stress resonates due to its impact on addiction [46]. Hyperpalatable foods (unhealthy foods that contain higher amounts of fat and sugar) have rewarding qualities. Therefore, stress may contribute towards the over-consumption of such unhealthy foods due to their addictive qualities [46]. Furthermore, stress may contribute towards ‘mindless’ eating due to distraction from eating behaviours, with associated changes in eating patterns and scenarios. Over time, such altered eating behaviour can result in altered neurobiology (including changes in hypothalamic neuropeptides and appetite-related hormones), with increasingly compulsive behaviour [46]. Chronic stress may also impact the mesolimbic dopaminergic system. The stress-related neurobiological changes outlined here may synergise to enhance reward sensitivity and preference for hyperpalatable foods, and result in physiological changes that increase fat mass [46]. 

Further research is required to explore how the mechanisms outlined here linking stress with neurobiological and physiological changes and the resultant increase in fat mass vary between individuals and the factors that may influence such mechanisms and their heterogeneity. Furthermore, individual stress responses to the same stressor may vary considerably between individuals. Therefore, use of the term ‘de-stress’ as a pillar of lifestyle is unhelpful, given that it is impossible to provide unifying advice in this regard. Furthermore, the elimination of stressors from our daily lives would be a naïve suggestion, and it seems logical to posit that however we live our lives, we will all experience stress. However, although many of our daily experiences are beyond our own control, there are useful ways in which we can deal with stress to minimise its harmful impact on us and our propensity for weight gain. In our own obesity service, we innovated and implemented mindfulness approaches (provided by obesity-focused dieticians and psychologists) to improve eating behaviours, and in the process improved self-esteem and self-confidence [35]. We also demonstrated sustained weight loss and improved engagement with clinic attendances over the longer term [47]. Our data demonstrate proof of concept that the application of mindfulness techniques directed towards improved eating behaviours manifest holistic attitudinal, emotional, and behavioural benefits. Such an approach could be improved with apps designed for mobile electronic devices [48] to remind people perhaps to take short breaks throughout the day to enable more accurate perceptions of their own internal requirements.

As a future approach, perhaps we could extend mindfulness techniques to enable their integration into all aspects of daily life (without a need for them being a separate item on the daily schedule), including, for example, how one works and enjoys leisure and family time. This approach would better equip people to deal with the stresses of everyday life, and thereby to protect our vulnerable neurobiological and hyperpalatable food addictive responses. Future studies should further explore the effects of mindfulness [49] and other techniques applied in diverse settings, such as ‘Jacobson’s Progressive Muscle Relaxation’ [50], yoga [51] or ‘autonomous sensory meridian response’ [52], to improve our handling of stress.

### 3.5. Optimising Social Conditions

The final pillar of lifestyle is one which is less directed at the individual and more directed at the fabric of society, and in particular governments, regulators, food companies and retailers. Given the disparity in food prices for healthy versus unhealthy foods, especially in the current crisis in the cost of living, more should be done to ensure that healthy foods are affordable even for the poorest members of society, who often have the greatest need for a healthy diet. Furthermore, we need societal change to promote rather than stigmatise healthy foods. This could be done through advertising, for example, including a restriction on advertising unhealthy foods generally and particularly to our children. More needs to be done to educate the populace on healthy diets, including for people with mental health problems (a strong and common risk factor for obesity), mandatory, clear and transparent food labelling for ultra-processed foods (UPFs), and less of on an emphasis on the caloric content of food, but rather the optimisation of a balanced diet with close attention to micronutrients. Governmental advice should avoid a ‘one-size-fits-all’ approach to recommended (and sex-specific) caloric intakes, and instead recognise that healthy diets are uniquely individual and focus more on generalities that apply broadly across the populace (the focus of this review article). Employers should ensure adequate leisure time, including sufficient breaks for eating, and an option for employees to purchase and consume healthy food. Regarding healthcare professionals who offer dietary advice to patients, there needs to be better overall regulation and quality certification, and a greater emphasis on diet within the medical school curriculum. Finally, food companies need to act more responsibly, and there should be more regulation to restrict the production of foods that elicit a highly hedonic and addictive response purely for profit generation for nutraceutical companies, but at the expense of causing negative health outcomes, multi-morbidity and premature mortality for millions of people. It seems illogical that our drug therapies are so highly regulated with safety as a key concern, and yet when it comes to our diet and the regulation of the foods that we eat, our regulators seem relatively lackadaisical and unconcerned, seemingly complicit with the mindset of many food companies to prioritise profit over social responsibility. For the future health and wellbeing of humankind, this must change. Now.

## 4. Balancing Macronutrients

Having built the foundations for dietary modification through the establishment and maintenance of four key pillars of lifestyle, we turn our attention to balancing dietary macronutrients. In our highly westernized diets, replete with UPFs, which are often carbohydrate-laden and fibre-impoverished, the need for balancing dietary macronutrients has never been more pressing. It is worth reflecting on the role of our modern-day food environment and its mismatch with our genetic endowment, that has created an urgent need for macronutrient balance. We evolved to eat as much we can get (instead of a predilection for specific food types as proscribed by the ‘palaeolithic diet’), in times of limited food supply, predisposing to weight gain and obesity in times of food excess. There does not appear to be any ‘evolutionary’ diet as such. Healthy diets are those that establish and maintain health, and dietary requirements are likely to vary between individuals. Interestingly, ethnicities that have low dietary carbohydrate intake over prolonged periods, like Inuits, do not change their physiology and maintain their ability to digest carbohydrates [53]. Our digestive and metabolic systems evolved to function in response to the consumption of all contemporary foods, without immediate side effects. The longer-term metabolic and cardiovascular sequelae of a poor diet, with its associated premature mortality, have not provided a sufficient evolutionary filter for such unhealthy dietary behaviours. The human brain is designed to reinforce the intake of unhealthy rewarding foods under the Cro-Magnon assumption that food accessibility will inevitably be limited sooner or later. Furthermore, even seemingly healthy foods can, when consumed in excess, cause potential harm. This includes semi-toxic compounds such as phytic acid, oxalic acid or tannins (stealing minerals), lectins (dissolving erythrocytes), glucosinolates or flavonoids (being bitter), and unfermentable fibre (we are not ruminants). Still, we actively recommend foods abundant in these compounds that are potentially harmful when consumed in excessive amounts. 

There is a wealth of diet-related data in the medical literature, including topical reviews from our own group on topics such as the health benefits of the Mediterranean diet [54] and dietary influences on the gut–brain axis [55]. Whilst it is beyond the scope of this concise review to cover an exhaustive overview of the entire field, instead we focus on four key macronutrients that are often imbalanced in our diet but that need to be balanced to achieve micro-nutritional harmony: dietary fibre, carbohydrates, protein and ultra-processed foods (UPFs). 

### 4.1. Dietary Fibre

Recently, we published a detailed overview of the health benefits of dietary fibre [56]. Here, we provide a summary of those data. Dietary fibre is a non-digestible form of carbohydrate (lignin) with large molecular size (polysaccharides: ≥10 monomers) [57]. The European Food Safety Authority (EFSA) provide a long list of substances that constitute dietary fibre [57]. The current recommended dietary fibre intake for adults living in the US and in most European countries is in the range of 25–32 g per day for women and 30–35 g per day for men [58]. However, based on a comprehensive review of the published literature, dietary fibre intake for adults living in the Western world is around a third lower than that which is recommended [56,58]. By implication, adults living in the Western world should increase their dietary fibre intake by around 50% [56].

Scientific interest in the health benefits of dietary fibre was re-awakened in the 1970s [58,59]. Since then, much evidence supports an important role of dietary fibre in the regulation of metabolic health including insulin sensitivity, at least in the shorter term [60,61,62,63,64,65]. Dietary fibre intake may also improve glycaemic control, as evidenced by an observational report of a Japanese cohort without T2D, in which dietary fibre combined with an exercise program was associated with a reduction in HbA1C [66]. Furthermore, our own group published interventional data from the ProFiMet cohort in which we demonstrated a significant association between the ingestion of high cereal fibre and insulin sensitivity [67]. A systematic review and meta-analysis of the metabolic effects of dietary fibre and whole grains in the management of diabetes mellitus also demonstrated that the intake of a high-fibre diet is associated with improved insulin sensitivity and key indices of glycaemic and lipid control [68]. Although most fibre-laden foods contain both soluble and insoluble forms of fibre (including those discussed in the studies outlined here), it is important to note that soluble dietary fibre is associated with specific improvements in both lipid profiles and efficiency of assimilation of carbohydrate-rich foods [63,69,70]. However, most of the evidence that associates dietary fibre with long-term improvement of insulin sensitivity and reduced risk of developing T2D relates to insoluble forms of fibre, including whole grain products [56,71]. 

In addition to improvements in insulin sensitivity, there is also some evidence that dietary fibre is associated with modest improvements in body weight, although the reported studies are relatively short-term, and there is a notable absence of longer-term studies reported in the literature, with the notable exception of a rodent-based study [72]. A systematic review and meta-analysis of the literature showed that high-fibre diets consisting of dietary pulse interventions over 6 weeks were associated with an overall significant reduction in body weight of −0.34 kg [73]. A separate systematic review and meta-analysis of viscous fibre ingestion showed an association with a similar degree of weight loss of −0.33 kg [74]. Despite the relative lack of longer-term data exploring the effects of dietary fibre intake on body weight, and the underwhelming magnitude of weight loss in the shorter term, it does appear that intake of dietary fibre promotes weight loss and dietary adherence in adults with overweight or obesity in the context of a calorie-restricted diet (−750 Kcal per day) over 6 months, in the ‘Preventing Overweight Using Novel Dietary Strategies’ (POUNDS Lost) study [75]. In this study, as a predictor of weight loss, dietary fibre intake was superior to multiple other dietary and anthropometric factors, and was strongly associated with adherence to macronutrient prescriptions [75]. Interestingly, long-term rodent-based studies (over 67 weeks) with diets containing variable amounts of fibre (cellulose vs. guar gum) suggested that weight-loss effects and favourable metabolic effects of dietary fibre intake may only become manifest in the longer term [72,76]. However, such duration-dependent metabolic effects of dietary fibre have not been demonstrated conclusively in human-based studies. 

The mechanisms by which dietary fibre mediates its metabolic benefits are incompletely understood. However, it seems likely that at least some of these benefits are mediated through effects on the gut microbiota, a collection of around 100 trillion microbes that, over an unimaginable timescale, co-evolved with our hominid ancestors [77]. The gut microbiota have become topical in recent times, and seem essential for the normal development of our immune system, our metabolic health, and even our emotional and mental functioning [77]. Furthermore, much 21st century chronic illness with underlying immuno-inflammatory pathogeneses likely stem, at least in part, from gut dysbiosis. Our insights into the importance of dietary fibre and the gut microbiota for health and disease, including propensity to colitis, derive primarily from rodent-based models. This includes a gnotobiotic mouse model (with colonization from harvested human gut microbiota) in which host-secreted mucus glycoproteins were used as an alternate nutrient source for the gut microbiota, in the context of chronic dietary fibre deficiency [78]. Ultimately, this process led to erosion of the colonic mucus barrier and predisposition to lethal colitis [78]. In addition to the establishment and maintenance of the health and integrity of the intestinal barrier, dietary fibre also provides a milieu in which the gut microbiota form a healthy ecology. This enables the production of microbial metabolites (through, for example, the anaerobic fermentation of dietary fibre) that are key for our health and wellbeing, such as secondary bile acids, tryptophan metabolites [79,80] and short-chain fatty acids (SCFAs) [81]. SCFAs appear to provide numerous health benefits, including an energy source for colonocytes [56], facilitation of gut motility [82], G protein-coupled receptor-mediated effects on the metabolism of glucose, lipids, and cholesterol [83], and improved insulin sensitivity and appetite suppression [84]. Such metabolic benefits of SCFAs have been confirmed by data from human-based studies [85]. In addition to these peripheral effects, SCFAs may also act centrally (following translocation across the blood–brain barrier) to enhance hypothalamic appetite suppression [84] and regulate other metabolic pathways [86,87]. 

Although there are multiple potential mechanisms that mediate the metabolic benefits of dietary fibre, it seems likely that their effects on the gut microbiota are important. Fortunately, our gut microbiota are modifiable through changes to our diet and lifestyle [77]. The relative lack of dietary fibre in Western diets likely contributes towards metabolic dysfunction and modern-day chronic illnesses. There are many explanations for our modern-day fibre-impoverished diets, including a general lack of fibre in many UPFs, a relative lack of plant-based food in our diets, and the higher cost of high-fibre, unprocessed, and fresh foods compared with UPFs [88,89,90]. Furthermore, some people may simply not tolerate a high-fibre diet. Prior to the final section, in which we outline a suggested strategy for dietary modification, we outline evidence to support a reduction of dietary carbohydrate intake, optimisation of protein intake, and limitation of the consumption of UPFs.

### 4.2. Carbohydrates

One problem associated with studying the metabolic effects of the low-carbohydrate diet (LCD), or any dietary strategy, is the impossibility of studying the effects of that dietary intervention in isolation. Inevitably, dietary restriction of any micronutrient requires substitution of the deficient micronutrient with an alternate micronutrient. Furthermore, the LCD may result in subtle changes to food preferences, particularly an aversion to high-fat foods (given our predilection for the entirely unnatural and artificial combination of fat and sugar in our foods). Indeed, such a preference for low-fat food options in some people following the LCD may account for some of its metabolic benefits. Furthermore, the LCD is often accompanied by a compensatory increase in dietary protein intake. With these caveats in mind, we recently published a detailed overview of the metabolic benefits and potential limitations of adopting an LCD [91]. It is generally accepted that a key factor underlying the growing global obesity problem in the last half-century has been the encouragement and recommendation of low dietary fat intake [92]. This has driven an excessive intake of simple carbohydrates and sugar in our diets (high-carbohydrate diet [HCD]), primarily through sugar added to our highly processed foods [92], to improve palatability. Although food companies would indubitably argue that the sugar added to UPFs is required to ensure their palatability (and therefore acceptability to the consumer), the sheer amount of sugar added to many UPFs strongly suggests an ulterior motive to trigger the hedonic and rewarding neural circuits with the inevitable addictive qualities of UPFs, thereby ensuring the profitability of UPFs. 

To understand the metabolic benefits of an LCD, it can be instructive to explore the adverse metabolic consequence of adopting an HCD, including the associated increased levels of serum insulin that partitions energy towards storage depots, including fat deposition in adipose tissue, and away from oxidation [93]. Elevated levels of insulin have central hypothalamic effects as a potent anorexigenic hormone (with insulin receptors widely distributed throughout the central nervous system) [94], and may also suppress the overall metabolic rate [93]. Furthermore, an HCD can also elicit hedonic pleasurable effects that drive us to eat more [91]. The adverse metabolic consequences of an HCD, and the associated promotion of fat deposition and weight gain, provides an important rationale for advocating the LCD as a key dietary strategy for obesity. 

The LCD consists of <130 g of carbohydrate per day [95], or a diet in which carbohydrates form <20% of the total daily calories [96]. A key metabolic benefit of the LCD is the associated reduction in the hepatic conversion of excessive carbohydrates into fatty acids, and concurrent enhancement of lipolysis [91]. Furthermore, reduced serum levels of insulin result in a diminished drive to store fat in adipose tissue, manifesting in reduced fat mass [93]. In a meta-analysis of 14 randomized controlled trials (RCTs) with the inclusion of >1400 individuals living with obesity, it was shown that those adopting an LCD or very low-carbohydrate diet (VLCD) had a greater reduction in fat mass by 0.77 kg compared with those who adopted a low-fat diet (LFD), and over 12 months, those who adopted the LCD still had an additional reduction in fat mass of 0.57 kg [97]. These data demonstrate proof of concept that, at least over the relative short term, the LCD can represent an effective dietary strategy for weight loss in obesity. However, it should be acknowledged that the LCD, particularly over the longer term, does have some limitations and potential safety concerns, relating primarily to its ketotic and nutritional effects [91]. Furthermore, some epidemiological studies—with their various methodological limitations—report an association between adoption of the LCD and increased mortality [98]. One unfortunate consequence of the LCD is the potential for a diminished supply of glycogen for muscles and liver, that may then contribute towards fatigue and impaired ability to engage in physical and athletic activities [99]. Furthermore, adoption of the LCD can result in reduced pleasure from eating and impaired food-related social interactions, that in turn can negatively impact mental and emotional health [91]. Widespread adoption of the LCD may also have negative ecological and environmental consequences (from increased demand for meat production and associated deforestation and climate change) [91]. Finally, adoption of the LCD can have socio-economic implications given its relative expense compared with other diets, which may preclude its adoption by people from lower socio-economic groups [100].

Despite these caveats, it is important to note that in addition to an overall reduction in fat mass, the LCD, at least in the short-term, provides other metabolic benefits that include improvements in fasting insulin sensitivity and glycaemic control (from a diminished supply of carbohydrates to the liver and reduced levels of serum insulin) [91]. However, the impact of the LCD on cardiovascular risk and outcomes over the longer term is contentious. Although in patients with T2D and pre-diabetes, adoption of the LCD may reduce the future risk of cardio-vascular disease [101,102], as outlined earlier, the only published cohort study to date that investigated the impact of long-term LCD adoption on cardiovascular disease outcomes showed an increase in mortality, with increased serum levels of low-density lipoprotein (LDL) cholesterol (low atherogenic, large particle LDL-cholesterol) [98]. Furthermore, the only long-term RCT on the LCD (PREVIEW) that investigated the incidence of diabetes mellitus in pre-diabetes participants (with randomisation to High Protein-LCD versus Moderate Protein-LFD) showed no difference between these two diets [103]. Although PREVIEW demonstrated a low overall incidence of diabetes mellitus due to an intensive first-phase weight-loss in all the groups, this study cannot support the superiority of the LCD over the LFD regarding prevention of the development of diabetes mellitus [103]. Furthermore, the weight-loss effects and metabolic benefits of the LCD, such as improved fasting insulin sensitivity, tend to diminish over the longer term [101,102]. Finally, given the origin of carbohydrates in plant-based foods such as legumes and vegetables, one concern of adopting the LCD is that this may restrict intake of essential micronutrients such as polyunsaturated fatty acids (PUFAs) and fibre, which in turn may worsen mortality [104,105]. 

To conclude this subsection, adoption of the LCD may be a reasonable dietary strategy for obesity management but only in the shorter term, particularly in the context of T2D or pre-diabetes. Such an approach will likely improve fasting insulin sensitivity and fat mass. However, as a longer-term strategy, the adoption of a plant-based diet (that typically contains 50–55% carbohydrates) is important to ensure adequate intake of dietary fibre and essential micronutrients. 

### 4.3. Protein

Although beyond the scope of this concise review, it is important to appreciate the benefits of a high-protein diet. In addition to satiating effects [71], boosting of energy expenditure and enhanced hepatic lipid metabolism [106], a high-protein diet also helps to build and sustain muscle mass, which in turn enhances metabolic efficiency and resting energy expenditure [107]. These effects implicate multiple metabolic pathways, including the release of incretin hormones from the gastrointestinal tract, blood amino acids and changes in serum leptin levels [108]. Such metabolic effects of high-protein diets can result in reduced food intake and improved body weight and adiposity over the longer term [108]. In an excellent systematic review and meta-analysis of randomized controlled trials, it was concluded that compared with lower protein diets, higher protein diets were associated with small but favourable effects on weight loss and other benefits that included some loss of fat mass, improved lipid outcomes, and reduced systolic blood pressure [109].

Despite the clear benefits of a high-protein diet outlined here, there are some potential health concerns, that stem from rodent-based data, regarding the longer-term effects of high-protein diets that implicate skeletal and renal physiology [108,110,111]. However, existing data on the potential health concerns of a high-protein diet in humans (particularly in the context of renal dysfunction) are inconclusive and contentious, with limited data on health-related quality of life, possible adverse effects [112], and compliance with protein-restricted diets in renally impaired people [113]. Any dietary advice for patients with renal dysfunction needs to be tempered with the possibility that over-restriction of protein intake may lead to adverse consequences that include increased propensity for malnutrition [114] and worsened immune function. Indeed, over the longer term, the restriction of dietary protein intake has uncertain effects on renal functioning [113]. Regarding the health effects of dietary protein in patients with diabetes mellitus, a guideline review suggested that the range of protein intake appears safe and adaptable to the dietary preferences of each individual [115]. It is important that future studies address the current uncertainties regarding the health benefits versus potential risks of the adoption of a high-protein diet in humans, to enable clear and unambiguous guidance for dietary management in the context of renal impairment. This guidance should be based on evidence from long-term randomized controlled trials with participants recruited at different stages of chronic kidney disease [113]. Such trials should also explore the relative anabolic benefits of plant- vs. animal-based proteins in this context [116].

Finally, it is important to consider the possible impact of a high-protein diet on carbohydrate intake and overall risk for cardiovascular disease (CVD). In one study, it was shown that most ‘Plant-Based Alternative Products’ (PBAPs) had a superior nutrient profile compared with animal-based homologs [117]. Furthermore, due to their high degree of processing and variations in nutritional profiling, PBPAs require a multi-dimensional assessment of their overall health impact [117]. However, despite these caveats, PBAPs may facilitate the restriction of dietary carbohydrate ingestion. In a recent analysis of the UK biobank cohort on the cardiovascular disease (CVD) implications of plant-origin foods based on their processing, it was shown that non-UPF plant-based food was associated with improved CVD risk, whereas UPF plant-based food increased risk of CVD [118]. Therefore, the role of food processing is an important consideration for CVD outcomes, even for plant-sourced diets. Importantly, age may influence the CV effects of plant-based foods, as this study included more participants with older age and higher CVD risk, who may respond differently to diet with a high content of PBAPs compared with younger, healthy non-smokers [119]. Although the longer-term adoption of a plant-based dietary approach does not meet the strict definition of an LCD, it is still important to restrict overall intake of dietary carbohydrates through reduced intake of ultra-processed foods that often contain large quantities of sugar.

### 4.4. Ultra-Processed Foods (UPFs)

UPFs are industrially formulated and derived from natural food or synthesized from other organic compounds. Due to their industrial formulation, UPFs cannot be recreated in the home kitchen. The precise definition of UPFs has varied over time, and due to the difficulty of interpreting such definitions, the NOVA (‘nova classificação’; new classification) group has provided lists of examples of foods that should be classified as ultra-processed [120]. In recent times, there has been much scientific focus on UPFs regarding their nutritional quality and as a risk factor for the development of noncommunicable diseases [120]. There are important limitations of NOVA and NutriScore; for example, NOVA renders a food as ‘level 4’ (equivalent to UPF), even if just one single additive is added (e.g., one sweetener, preservative or colour agent to an otherwise simply cooked, frozen, canned or mashed food). Foods that are NOVA-classified as ‘level 4’ require greater distinction. Furthermore, the NOVA classification of ‘level 2’ foods can be misleading (particularly for salt and fats). As an example of the limitations of the NOVA food classification, packaged tofu or seitan is NOVA-classified as ‘level 4’, but basically consists of condensed plant protein with low amounts of fat, and from a health perspective has similarities to plain sirloin steak (‘level 1’). Many healthy plant-based foods, such as soy yoghurt, are classified as highly processed, and the classification therefore does not always capture the health aspects of foods. Based on the NOVA classification, some ‘UPFs’ are healthy. Furthermore, the NutriScore provides evaluations for specific food groups (e.g., frozen pizzas), focussing on contents of fat, carbohydrates, fibre, and salt, but not the quality of those fats and carbohydrates, or the addition of preservatives and sweeteners. Therefore, the NutriScore can be misleading (e.g., a NutriScore of ‘A’ could be applied to a junk food pizza). A similar problem exists for the glycaemic index (GI) of foods. As a general guide, most UPFs and high-GI foods are unhealthy, but not all. Conversely, most NOVA level 1–2 and low-GI foods are healthy, but not all. Due to the increased consumption of ‘ready-made’ foods, it is important that the quality of such UPFs is improved to optimise their healthiness. 

It is beyond the scope of this concise review to provide an exhaustive discussion of this topic, but rather, we provide an overview of the metabolic hazards of ingesting a diet predominant in UPFs, including impact on weight gain and obesity. We use this insight as a rationale for promoting dietary restriction of ultra-processed foods as a key strategy for obesity management. This aligns with the public health nutrition advice of NOVA to avoid UPFs to improve the intake of nutrients, emphasising sugar, fat and salt [120]. Importantly, UPFs also tend to be impoverished of dietary fibre [120], providing another key rationale for recommended restricted intake.

Some of our most compelling evidence for the adverse metabolic effects of ingesting UPFs stems from reported systematic reviews and meta-analyses of the current literature, including one that explored the effects of UPFs on noncommunicable disease risk, morbidity, and mortality, including 43 observational studies on >891,000 participants [121]. In this review, it was demonstrated that the consumption of UPFs is associated with increased risk of overweight (OR 1.36), obesity (OR 1.51), abdominal obesity (OR 1.49), metabolic syndrome (OR 1.81), and all-cause mortality (hazard ratio 1.28). There was also an association of UPF ingestion with depression (hazard ratio 1.22) and other pathologies that include some cancers, irritable bowel syndrome and functional dyspepsia [121]. Furthermore, there was an association between the ingestion of UPFs and the development of metabolic syndrome in adolescents and dyslipidaemia in children [121].

Unfortunately, UPFs are ubiquitous in our Western diets. Supermarkets have whole aisles stacked with UPFs. Our food environment is dominated by UPFs, and reciprocally, there is restricted availability of healthy unprocessed foods. This is coupled with the relative cheapness of UPFs and the convenience of reduced cooking times, particularly for time-pressured people who may need to balance the demands of work, family, and child rearing into daily life. It is also understandable why supermarkets and consumers may prefer UPFs due to their relative affordability, increased shelf-life, improved transportability and versatile stacking and storage options. Indeed, aspects of our modern society and infrastructure only function because of UPFs, including their relatively long shelf-life, safety from microbial contamination, and favourable digestibility (lectins, fibre, shells). However, despite these benefits, it is important to appreciate the harmful health effects of ultra-processed foods, and particularly for this discussion, their association with weight gain and obesity. Some of our best evidence stems from an in-patient RCT of ad libitum food intake in 20 weight-stable adults, randomised to either UPF or unprocessed food diets for 2 weeks immediately followed by the alternate diet for 2 weeks [122]. Energy intake was greater for the UPF diet, with increased consumption of fat and carbohydrate, but not protein, and weight changes aligned with energy intake (either gain or loss of 0.9 kg body weight for the UPF and unprocessed food diets, respectively) [122]. 

In addition to their association with excessive energy intake and consequent weight gain, a relative lack of key micronutrients in UPFs, including their impoverishment of dietary fibre, is a key concern. Given their ubiquity in our modern-day food environment, it would perhaps be unreasonable to expect total avoidance of UPFs in our diet. However, as an important dietary strategy both for the treatment and prevention of obesity, it is essential to restrict our dietary intake of UPFs. Furthermore, food industries should be encouraged to fortify UPFs not just to improve their palatability, but crucially to optimise their key micronutrient content, including dietary fibre. 

## 5. Suggested Dietary Strategy for Obesity

**Figure 1:**
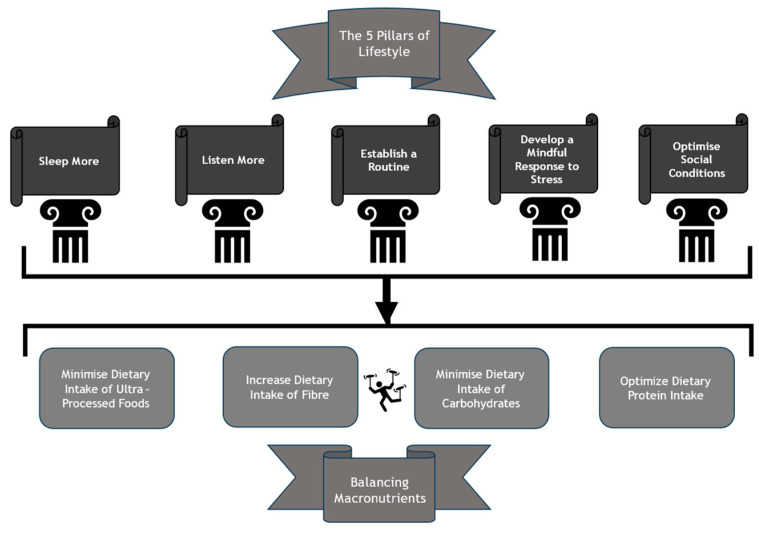
Suggested Dietary Strategy for Obesity.

Having discussed the foundations of dietary modification and the importance of balancing key macronutrients in our diet, here we outline a suggested strategy for dietary modification to be applied as an integral part of effective management of obesity (Figure 1):

***Sleep more:*** Most of us are sleep-deprived. Given the importance of sleep for our physiology and our appetitive and metabolic function, we should all prioritize our sleep. As adults, the recommended sleep duration is 7.5 h per night. We should establish a regular sleep–wake routine wherever possible and avoid any distractions (such as mobile electronic devices) during the protected time of sleep. We should ensure good sleep hygiene, and where possible, engage in regular physical activity during the day (but avoid this in the 2 h before sleep onset) and ensure our sleeping environment is dark to help to optimise restful and plentiful sleep. Where relevant, sleep-disordered conditions such as OSA should be diagnosed and managed to optimize both sleep duration and quality.

***Listen more:*** Our world is full of distractions to consciousness and subconsciousness. However, we need to learn to listen to our own signals, including hunger, and to respond appropriately. Adopting a more mindful approach to eating behaviours can work very well and act as a guide for us to listen more to the signals within, rather than to behave mindlessly. Within this paradigm, it is useful to only engage in eating behaviour at times of hunger, and to refrain from eating for any other reason. This may mean skipping occasional meals and avoidance of grazing, snacking and eating for emotional reasons.

***Establish a routine:*** Our lives are chaotic. We often need to juggle many balls concurrently. For many of us, establishing a routine is challenging. However, a routine will help to regulate a healthy sleep–wake cycle and help to ensure that we engage in time-appropriate behaviours. To help facilitate and reinforce such a routine, it can be useful to punctuate daily activities. For those who work from home, for example, dividing work time with leisure time in the same home environment could be punctuated by a brisk walk outside. Furthermore, only using the bed during the sleeping period can help to reinforce signals to the brain for maintenance of routine. 

***Develop a mindful response to stress:*** Stress is an inevitability of modern-day life. We cannot avoid stress. However, we can control to some extent how we respond to stress. If unchecked, stress can lead to important neurobiological changes that result in cravings for unhealthy hyperpalatable foods. There are many ways in which we can respond optimally to stress, and these are likely to vary for each of us. It is beyond the scope of this review to overview these. However, mindfulness approaches may help to focus the mind on what is important to us, and to regulate our emotional (and appetitive) responses to stress. This in turn can help to control our behavioural response to the experience of stress.

***Optimise social conditions:*** On a societal level, we need far better regulation of our food companies through updated legislation that ensures an approach that is socially responsible rather than profit-oriented. In short, our UPFs need to be produced with a strong emphasis on health, with mandatory requirements to ensure that they contain micronutrients such as dietary fibre, of which many of us living in the Western world are impoverished. For the health and wellbeing of humanity, this essential action and change of mindset is needed now. 

***Increase intake of dietary fibre:*** As outlined, there are multiple health benefits to dietary fibre. In essence, we need to increase the plant-based component of our diet, through vegetables, fruits, nuts, beans and pulses. For many years now, governments have promulgated the importance of ‘5-a-day’ portions of fruit and vegetables. Whilst this may be helpful for some of us, the key message is that we need to eat a varied diet, with our plant-based foods from as diverse a provenance as possible. Through focusing on optimising our plant-based diet, we will ensure that we eat dietary fibre in sufficient quantity to optimise our health and help protect us from much 21st century chronic illness. 

***Reduce dietary intake of carbohydrates:*** Over the shorter term, restriction of dietary carbohydrates can facilitate weight loss and have other favourable effects on metabolic health. However, this approach is limited in the longer term. Although not at all meeting the definition of an LCD, a plant-based diet typically contains 50–55% carbohydrates and ensures optimised intake of dietary fibre and other essential micronutrients. A general restriction of carbohydrates is a useful dietary strategy for obesity, but one that needs to be tempered over the longer term with plentiful provision and intake of fresh plant-based foods. 

***Optimize dietary protein intake:*** Although the human-based data for protein intake are less well-established than those for dietary fibre and carbohydrates, it is important to optimise our dietary protein intake, particularly that derived from plants (even those healthy plant-based foods that are processed and are classed as ‘UPFs’ by the NOVA classification). 

***Limit dietary intake of unhealthy UPFs:*** The ubiquity, convenience and relative cheapness of ultra-processed foods means that for many of us, it is challenging to restrict them in our diet. However, ultra-processed foods contain excessive quantities of sugar and salt and are impoverished of dietary fibre. Furthermore, ultra-processed foods lack essential micronutrients that are key to health and are present in fresh plant-based foods. As a key dietary strategy for obesity, it is important that we restrict our intake of ultra-processed foods and replace them with fresh plant-based foods to limit carbohydrate intake and optimise the intake of dietary fibre and other essential micronutrients. 

## 6. Concluding Remarks

Obesity stems from a complex interplay between our underlying genetic architecture and obesogenic environment. Media portrayal of obesity often focuses predominantly on the latter to a level whereby obesity can be categorised erroneously as a condition of lifestyle, and one that develops of our own choosing. Such a naïve perspective likely underlies our dispassionate response to obesity as a society, and our relative lack of empathy compared with other diseases such as malignancies (many of which, incidentally, are also heavily influenced by environmental factors and behaviours and obesity). The role of our genetics in the development of obesity is often overlooked. To compound this problem, eating behaviour is widely perceived to be under exquisite self-control and at the behest of our conscious willpower. This erroneous perspective is analogous to suggesting that our breathing is subject to continuous self-control. There are interesting parallels between eating and breathing in that both are controlled by sub-conscious neural pathways, but each can be countermanded by conscious control, at least *in the short term*. The important point, though, is that even during such conscious countermand, the subconscious signals remain and ultimately control both eating and breathing behaviour in the longer term. Therefore, rather than denial of hunger satiation, we should accept that we need to eat when we feel hungry and strive to satisfy our appetite for food accordingly, but crucially modify our eating behaviours and macronutrient intake in the ways outlined. In other words, we should adopt a healthy dietary strategy rather than leaving ourselves feeling hungry. The underlying genetic differences between individuals ensure that our predisposition to weight gain and our propensity to lose weight through dietary strategies are unique to each of us. In this review, we provide general principles for healthy dietary strategies in obesity. In our clinical practice, we need to apply bespoke dietary strategies that are tailored to each individual patient according to their specific needs. Furthermore, we need to refrain from judging the utility of a diet based simply on its associated magnitude of weight loss, but rather adopt a more holistic perspective in which a dietary strategy is valued for its overall health benefits, including the nurturing of our gut microbiota, to enable them to nurture and protect us. 

## Figures and Tables

**Table 1:** Summary of the dietary and lifestyle strategies for obesity.

Strategy	Modern-Day Problem	Impact on Propensity for Weight Gain and Obesity	Potential Solution
Sleep	Deprivation	Enhanced appetite	Need for adults to get 7–8 h sleep per night
Listening	Distractions; Relative deafness to internal and external signals	Appetitive dysregulation; Mindless eating	Actively listen to internal (appetitive) signals; Eat to hunger
Routine	Busy, chaotic and unpredictable lives	Dysregulated sleep–wake cycle; Predisposition to disturbed and mindless eating behaviours	Establish and maintain regular sleep–wake cycle; Individualize lifestyle recommendations
De-stressing	Stress as an inherent component of modern-day life	Mindless eating and over-consumption of hyper-palatable foods; Altered neurobiology with increasingly compulsive behaviours	Implement techniques to alleviate response to stress (such as mindfulness or yoga) to improve healthy eating behaviours
Optimize Social Conditions	Profit-driven and irresponsible food companies; Poor governmental control and regulation of our nutrition; Stigmatization and expense of healthy foods	Unaffordability of healthy foods; Easier choice of cheaper, unhealthy foods with hedonic and addictive effects	Implement much stricter controls and regulation of food companies; Government input to improve affordability of healthy foods
Dietary Fibre	Impoverishment	Plethoric negative impact on health and gut microbiota; Worsened CV and inflammatory outcomes	Improve dietary fibre intake generally; Increased availability and consumption of plant-based foods; Added fibre to UPFs
Carbohydrate	Over-consumption	Weight gain; Predisposition to T2D and other CV RFs	LCD in shorter term; Generalized restriction of carbohydrate intake in longer term
Protein	Relative restriction of plant-based protein intake	Reduced muscle bulk; Reduced metabolic efficiency and appetite control	Increase availability and consumption of plant-based protein
UPFs	Ubiquitous availability and over-consumption	Weight gain; Development of Metabolic Syndrome; Increased mortality	Restrict consumption of UPFs; Food companies to fortify UPFs with key micronutrients and fibre

(CV: Cardiovascular; LCD: Low-Carbohydrate Diet; RFs: Risk Factors; T2D: Type 2 Diabetes Mellitus; UPFs: Ultra-Processed Foods).
